# Malnutrition-Inflammation Complex Syndrome With Associated Atherosclerosis and Chronic Kidney Disease: A Case Report

**DOI:** 10.7759/cureus.23629

**Published:** 2022-03-29

**Authors:** Lakshmi Kannan

**Affiliations:** 1 Nephrology, Pikeville Medical Center, Pikeville, USA

**Keywords:** chronic kidney disease, atherosclerosis, hypoparathyroidism, inflammation, malnutrition

## Abstract

Patients with chronic kidney disease or on maintenance dialysis usually have secondary hyperparathyroidism. It is extremely rare for these patients to have hypoparathyroidism. It can be occasionally seen in patients on maintenance dialysis or after transplant if they have undergone parathyroidectomy for tertiary hyperparathyroidism or advanced secondary hyperparathyroidism. Also, the results of hypoparathyroidism on serum phosphorus in these patients have been infrequently reported in the literature. Here, we present a patient with chronic kidney disease stage 3 who presented with hypophosphatemia and hypoparathyroidism from the malnutrition-inflammation complex syndrome.

## Introduction

Renal osteodystrophy is common in patients requiring maintenance dialysis treatments. The spectrum of disorders includes high turnover bone diseases from active vitamin D deficiency and secondary hyperparathyroidism and low turnover bone disease from low parathyroid hormone levels (PTH) [1]. Low PTH levels are rarely seen in these patients’ factors, leading to low serum parathyroid hormone concentration, including hypercalcemia, ingestion of vitamin D products or active vitamin D analogs, and /or calcium-sensing receptor antagonists [2]. Another important factor is seen in the setting of malnutrition-inflammation complex syndrome (MICS) [3]. Although the association between high serum PTH (>300 pg/ml) and increased mortality has been shown, the association between low PTH, chronic inflammation, cardiovascular disease, and mortality has not been well-studied. We present a 72-year-old Chinese male who presented with chronic kidney disease stage 3, persistent hypoparathyroidism and hypophosphatemia, and frequent hospitalizations for atherosclerotic heart disease and in-stent stenosis.

## Case presentation

A 72-year-old Chinese male with a body mass index (BMI) of 17 kg/m^2 ^and medical history significant for hypertension, hyperlipidemia, type 2 diabetes mellitus, coronary artery disease post-coronary artery bypass graft (CABG) in 2012, obstructive sleep apnea, and chronic kidney disease (CKD) stage 3a was first evaluated by Nephrology in 2019 for his CKD. On initial evaluation, serum creatinine was 1.4 mg/dL with glomerular filtration rate (GFR) of 48 ml/min/1.73 m2. CKD was attributed to underlying diabetic nephropathy. In 2019, he was found to have low phosphorus at 2.6 mg/dL and a relatively low parathyroid hormone level of 13 pg/ml with normal calcium of 9.6 mg/dL and a vitamin D level of 32 ng/mL. The rest of the laboratory workup is shown in Table 1.

**Table 1:** Laboratory workup at the time of initial Nephrology visit NA: Not available.

Laboratory test	Reference range	2019
Hemoglobin	11-17 mg/dL	10.1
Hematocrit	32-51.6%	31
White blood cells	3-11.3 K/µL	13.6
Platelets	134-412 K/µL	304
Serum glucose	70-110 mg/dL	116
Serum sodium	133-144 mmol/L	138
Serum potassium	3.6-5.2 mmol/L	4
Serum calcium	8.3-10.4 mg/dL	9.6
Serum magnesium	1.7-2.4 mg/dL	2.1
Serum bicarbonate	21-32 mmol/L	23
Serum phosphorus	3.5-5.0 mg/dL	2.6
Serum uric acid	3.5-7.2 mg/dL	NA
Blood urea nitrogen	6-24 ng/dL	29
Serum creatinine	0.60-1.10 mg/dL	1.4
Estimated glomerular filtration rate	(>60 mL/min/1.73m2)	48
Serum albumin	3.4-5.4 mg/dL	2.9
Serum ionized calcium	1.19-1.29 mmol/L	NA
Serum aspartate transaminases	15-37 U/L	8
Serum alanine transaminases	12-78 U/L	18
Serum alkaline phosphatase	44-147 U/L	146
PTH, intact	8.0-74.0 pg/mL	13
25 OH vitamin D	20-80 ng/mL	32
1,25-OH vitamin D	20 to 45 pg/mL	19.9
Hemoglobin A1C	<6.5%	7.1
Urine microalbumin, random	1.3-30 mg/dL	74
Erythrocyte sedimentation rate (ESR)	(0-20 mm/hr)	NA
C-reactive protein (CRP)	(0-0.3 mg/dL)	NA
Urine protein creatinine ratio	<0.2	0.7

The search for an immunological basis for his hypoparathyroidism was unsuccessful; the patient had not had any thyroid surgeries or radiation to the neck. Anti-thyroglobulin, anti-microsomal, anti-smooth muscle, anti-mitochondrial, and anti-parietal cell antibodies were negative.

Between 2019 and 2021, the patient has had two admissions for chest pain and acute coronary syndrome without persistent ST-segment elevation with left heart catheterization (LHC) showing 100% left anterior descending (LAD) occlusion, moderate disease in circumflex, and patent obtuse marginal and right coronary artery (RCA). He had a stent placed in the LAD the first time. During his second LHC, he had an occluded LAD stent, 60-70% occluded circumflex, and 100% occlusion on the RCA, and stents were placed in the RCA and LAD again.

## Discussion

Adynamic bone disease is a type of renal osteodystrophy characterized by reduced osteoblasts and osteoclasts, resulting in markedly low bone turnover. It is most commonly seen in patients on maintenance dialysis and rare in CKD patients on conservative treatment. Even in these patients, adynamic bone disease is characterized by high serum PTH levels. Potential causes of low serum PTH include diabetes mellitus where high concentrations of glucose suppress parathyroid hormone secretion [4] and studies have shown an association between poor glycemic control and lower intact PTH levels [5], the use of cinacalcet, which leads to a considerable decrease in measured serum PTH, and fall in serum calcium and phosphorus. In a study by Dukkipati et al. [6], low serum PTH, defined as levels <150 pg/ml was prevalent among non-blacks, Hispanics, and diabetics in 748 patients on maintenance hemodialysis who were observed for five years in South California.

Individuals with chronic kidney disease have poor survival mainly from cardiovascular risks. Atherosclerosis likely is due to the coexistence of hypertension, hyperhomocysteinemia, inflammation, malnutrition, increased oxidative stress, and hyperlipidemia. Also, there is a high prevalence of protein-energy malnutrition, and it has been shown to have greater mortality and morbidity [7]. These patients have significant wasting, which can cause malnutrition-inflammation complex syndrome [8].

There is an increased risk of atherosclerosis-related cardiovascular disease in these patients. The recurrent acute coronary syndrome without persistent ST-segment elevation in our patient could be related to MICS. In the study by Yamada et al. [9], they demonstrated the impact of malnutrition and inflammation on the derangement in the bone-cardiovascular axis based on a risk score composed of age, serum levels of creatinine, albumin, C-reactive protein (CRP), and BMI.

The first report of patients with hypoparathyroidism and hypophosphatemia was reported in 2014 [10]. Our patient exhibited protein-energy malnutrition and it is likely attributed to inadequate nutrition due to dietary restrictions, abnormal nutrient metabolism due to hypercatabolism, and increased nutrient loss with the stress of surgery and hospitalizations. Also, low PTH leads to decreased accumulation of adipose tissue, which ultimately results in protein-energy malnutrition.

No therapeutic strategies exist for the treatment of hypoparathyroidism in patients with chronic kidney disease, and there is a paucity of consensus on MICS in terms of the degree of severity and management. One of the proposed management strategies is targeting patient-oriented dietary recommendations - aiming to decrease the glycemic index of carbohydrates, increase the concentration of adiponectin, and decrease the absorption of indoles and amines to reduce the toxic load [11-12]. The consumption of fruit, vegetables, and legumes is closely associated with fiber intake, which has a beneficial effect on gut microbiota and improves peristalsis, and the basic pH of plant foods diminishes metabolic acidosis and can reduce the production of proinflammatory cytokines.

## Conclusions

In conclusion, patients with CKD have a high prevalence of chronic inflammation and malnutrition and a high prevalence of MICS. In turn, hypophosphatemia and hypoparathyroidism lead to protein-energy malnutrition, and these patients are at a high risk of complications, including accelerated atherosclerotic disease. One important intervention is to get nutritionists involved in the initial stages to improve malnutrition and correct the electrolyte derangements, and further studies are necessary to determine whether these interventions can decrease the incidence of cardiovascular-related events.
